# Reliability and validity of the electronic version of the Hopkins verbal learning test-revised in middle-aged and elderly Chinese people

**DOI:** 10.3389/fnagi.2023.1124731

**Published:** 2023-06-12

**Authors:** Lichen Jiang, Ming Xu, Shunyao Xia, Jiahui Zhu, Qi Zhou, Luoyi Xu, Chuan Shi, Daxing Wu

**Affiliations:** ^1^Medical Psychological Center, The Second Xiangya Hospital, Central South University, Changsha, Hunan, China; ^2^Institute of Mental Health, Peking University Sixth Hospital, Peking University, Beijing, China; ^3^NHC Key Laboratory for Mental Health, National Clinical Research Center for Mental Disorders, Peking University Sixth Hospital, Peking University, Beijing, China; ^4^Department of Psychiatry, Sir Run Run Shaw Hospital, Zhejiang University School of Medicine, Hangzhou, Zhejiang, China; ^5^Medical Psychological Institute, Central South University, Changsha, Hunan, China; ^6^National Clinical Research Center for Mental Disorders, Changsha, Hunan, China

**Keywords:** reliability, validity, Alzheimer’s disease, amnestic mild cognitive impairment (aMCI), computerized applications

## Abstract

**Background:**

The aging population is increasing, making it essential to have a standardized, convenient, and valid electronic memory test that can be accessed online for older people and caregivers. The electronic version of the Hopkins Verbal Learning Test-Revised (HVLT-R) as a test with these advantages and its reliability and validity has not yet been tested. Thus, this study examined the reliability and validity of the electronic version of the HVLT-R in middle-aged and elderly Chinese people to provide a scientific basis for its future dissemination and use.

**Methods:**

We included 1,925 healthy participants aged over 40, among whom 38 were retested after 3–6 months. In addition, 65 participants completed both the pad and paper-and-pencil versions of the HVLT-R (PAP-HVLT-R). We also recruited 42 Alzheimer’s disease (AD) patients, and 45 amnestic mild cognitive impairment (aMCI) patients. All participants completed the Pad-HVLT-R, the Hong Kong Brief Cognitive Test (HKBC), the Brief Visual Memory Test-Revised (BVMT-R), and the Logical Memory Test (LM).

**Results:**

(1) Reliability: the Cronbach’s α value was 0.94, the split-half reliability was 0.96. The test–retest correlation coefficients were moderate, ranging from 0.38 to 0.65 for direct variables and 0.16 to 0.52 for derived variables; (2) Concurrent validity: the Pad-HVLT-R showed a moderate correlation with the HKBC and BVMT-R, with correlation coefficients between total recall of 0.41 and 0.54, and between long-delayed recall of 0.42 and 0.59, respectively. It also showed a high correlation with the LM, with correlation coefficients of 0.72 for total recall and 0.62 for long-delayed recall; (3) Convergent validity: the Pad-HVLT-R was moderately correlated with the PAP version, with correlation coefficients ranging from 0.29 to 0.53 for direct variables and 0.15 to 0.43 for derived variables; (4) Discriminant capacity: the Pad-HVLT-R was effective in differentiating AD patients, as demonstrated by the ROC analysis with AUC values of 0.834 and 0.934 for total recall and long-delayed recall, respectively.

**Conclusion:**

(1) The electronic version of HVLT-R has good reliability and validity in middle-aged and elderly Chinese people; (2) The electronic version of HVLT-R can be used as an effective tool to distinguish AD patients from healthy people.

## 1. Introduction

According to a recent report by the United Nations (World Population Prospects 2022: 10 Key; United Nations, 2022), China is currently undergoing an aging process due to rising life expectancy and declining fertility rates. As people age, various cognitive functions, especially memory, tend to decline. Studies have shown that memory exhibits a decreasing tendency with age, starting around age of 50 and becoming more marked after age 70 (Xu et al., 1985). Episodic memory, which is the most age-sensitive of the long-term memory systems (Nyberg, 1996), typically develops rapidly in childhood but tends to decline in adulthood (Kausler, 1994; Schneider and Pressley, 2013), with more rapid declines in very old age (Singer et al., 2003).

The Hopkins Verbal Learning Test-Revised (HVLT-R; Benedict et al., 1998) is a popular neuropsychological test for episodic memory (Rabin et al., 2016), comprising 12 items. It is brief, multi-dimensional, easily administered, and well accepted test with no ceiling effect. Therefore, it has the potential to be an ideal assessment instrument for short-term memory assessments or series of tests. A number of studies have supported the reliability and validity of the HVLT-R, including evidence of the test–retested reliability (Benedict et al., 1998; Barr, 2003; O’Neil-Pirozzi et al., 2012; Shi et al., 2015), concurrent validity (Shapiro et al., 1999), convergent validity (Lacritz et al., 2001; Kordes, 2004), and discriminant capacity (Frank and Byrne, 2000; Foster et al., 2009; Lonie et al., 2010; González-Palau et al., 2013). The HVLT-R has been demonstrated to be suitable for different ethnic groups and individuals with significant cognitive impairment (Shi et al., 2012; Scott et al., 2020; Díaz-Santos et al., 2021), with high classification accuracy and effectiveness in evaluating healthy elderly populations (Ryan et al., 2021), patients with mild cognitive impairment (MCI; González-Palau et al., 2013; Hammers et al., 2022), Alzheimer’s disease (AD) patients (Shapiro et al., 1999; Gómez-Gallego and Gómez-García, 2019; Hammers et al., 2022), and dementia patients (Frank and Byrne, 2000; Hogervorst et al., 2002; Foster et al., 2009; Liao et al., 2019).

As the global population ages, memory decline and impairment become more common. Therefore, it is increasingly important to assess and monitor memory function to detect and intervene early. However, traditional paper-and-pencil tests can be time-consuming and challenging to administer remotely. In recent years, the development of electronic and web-based memory tests has provided a convenient and accessible solution for assessing memory function. For example, Lewandowsky et al. (2010) developed an electronic version of a working memory test battery that has been shown to be reliable and valid for assessing working memory function. Additionally, the Cognitive Assessment for Dementia, iPad version (CADi), developed in Japan for large-scale dementia screening, can be run on an iPad and includes immediate and delayed recall of three words (Onoda et al., 2013). The electronic and online tests not only provide a standardized and reliable way to assess memory function, but also have the potential to reach a wider population and enable more frequent monitoring of memory function over time.

However, concerns have been raised about the reliability and validity of electronic tests compared to their traditional counterparts. While some studies have shown that electronic tests have good reliability and validity and can be compared to their traditional paper-and-pencil counterparts (Hoskins et al., 2010; Vanderslice-Barr et al., 2011), other researchers have found that participants’ computer anxiety, familiarity with electronic devices, and motor coordination may affect the results of the test, and these factors may reduce the reliability of the electronic test compared to the paper-and-pencil version (Whitener and Klein, 1995; Barrigón et al., 2017). Therefore, when an electronic test is administered, the reliability and validity of the electronic version should be examined, rather than relying solely on the previous paper-and-pencil results.

Given the many advantages of electronic tests, a pad version of the HVLT-R has recently been developed, which can automatically read out the standardized instructions and records the answers. However, its reliability and validity are unknown. As such, the present study sought to examine the reliability and validity of the electronic version of the HVLT-R in middle-aged and elderly Chinese individuals, providing a scientific basis for its future use in research or clinical settings.

## 2. Methods

### 2.1. Participants

A total of 2,077 participants were recruited by convenience sampling in communities or clinics from 6 regions in China between May 2019 and June 2021. All subjects completed the Chinese Neuropsychological Consensus Battery (CNCB) in its electronic version (Wang et al., 2019). This study is part of the larger Chinese Neuropsychological Normative (CN-NORM) project led by the Dementia Care & Research Center, Peking University Institute of Mental Health (Sixth Hospital). All subjects and their families signed informed consent.

#### 2.1.1. Healthy sample

Subjects had to be over 40 years of age and were excluded if they scored <7 on the brief Community Screening Instrument for Dementia (CSI-D; Prince et al., 2011), had any medical condition that might interfere with cognition or had a related neurological disorder or major psychiatric disorder that could impact cognitive function (e.g., severe audiovisual impairment; history of drug or alcohol abuse; clear history of severe cerebrovascular disease (including cerebral hemorrhage and cerebral infarction), Parkinson’s syndrome, epilepsy, bipolar disorder, and other neuropsychiatric disorders; severe head trauma, carbon monoxide poisoning).

Depending on the administration procedure, we divided the healthy subjects into three groups as follows:

Sample 1: a total of 2,146 middle-aged and older adults completed the electronic version of the test battery. After excluding 221 subjects with a large number of missing values of the Pad-HVLT-R (e.g., incomplete short-delayed recall, long-delayed recall, or recognition trial), the final sample size was 1,925 people, resulting in a valid sample rate of 90%.

Sample 2: 44 subjects from Sample 1 were selected to complete the electronic version of the test battery again 3–6 months later to examine the test–retest reliability, of which 2 participants who did not complete the delayed recall were removed from analysis.

Sample 3: 66 subjects were administered both the electronic and PAP version of the test battery to examine the convergent validity. To counteract the sequential effect, a balanced design was used in this study. The 66 participants were divided into two groups, with the first group completing the PAP version of the test battery in the first phase and the electronic test later, while the second group did the opposite. One case was excluded for failure to complete the long-delayed recall of the Pad-HVLT-R. The interarrival time between two phases is shown in Table 1.

**Table 1:** Descriptive characteristics of three healthy sample.

Characteristic	Sample1	Sample2	Sample3
No. of participants	1,925	42	65
Age (years, mean ± SD)	57.77 (10.23)	59.29 (8.47)	58.48 (8.31)
Range	40–92	48–80	42–76
***n* (%)**			
40–49	471 (24.5%)	3 (7.1%)	11 (16.9%)
50–59	546 (28.4%)	19 (45.2%)	25 (38.5%)
60–69	682 (35.4%)	15 (35.7%)	22 (33.8%)
70–79	193 (10%)	3 (7.1%)	7 (10.8%)
≥80	33 (1.7%)	2 (4.8%)	-
Gender (male%)	43.2% (832/1093)	23.8 (10/32)	33.8 (22/43)
Education (years, mean ± SD)	9.21 (4.94)	8.57 (4.16)	8.33 (3.87)
***n*%**			
0–6	554 (28.8%)	12 (28.6%)	27 (41.5%)
7–9	531 (27.6%)	17 (40.5%)	19 (29.2%)
10–12	437 (22.7%)	9 (21.4%)	14 (21.5%)
13–16	391 (20.3%)	4 (9.5%)	4 (6.2%)
≥17	12 (0.6%)	-	1 (1.5%)
Interarrival time (days, mean ± SD) Range	-	112.07 (35.52) 65–217	118.62 (43.52) 68–236

#### 2.1.2. Clinic sample

A total of 100 subjects were enrolled, including 51 patients with amnestic mild cognitive impairment (aMCI) and 49 patients with AD. Among them, 6 aMCI patients and 7 AD patients failed to complete the immediate recall or delayed recall and were deleted. Finally, valid samples were collected: 45 aMCI patients and 42 AD patients. The details on the AD/MCI determination have been described in a previous study (Gu et al., 2023). Briefly, the participants completed a standardized neuropsychological assessment, underwent clinical interviews and brain imaging examinations, and received a clinical diagnosis by a memory specialist.

The inclusion criteria for patients with aMCI were: (1) Age ≥ 40; (2) Met the 2004 Petersen diagnostic criteria for aMCI (Petersen, 2004); (3) Can understand instructions; (4) Hachinski Ischemia Scale ≤4; (5) Geriatric Depression Scale ≤10.

The inclusion criteria for patients with AD were: (1) Age ≥ 40; (2) Met the diagnostic criteria of “probable AD dementia” of the National Institute on Aging-Alzheimer’s Association (NIA-AA, 2011; Jack et al., 2011); (3) Can understand instructions; (4) Hachinski Ischemia Scale ≤4; (5) Geriatric Depression Scale ≤10.

The exclusion criteria for both aMCI and AD patients were: (1) Unwilling to cooperate; (2) Severe audiovisual impairment; (3) History of drug or alcohol abuse; (4) A clear history of severe cerebrovascular disease (including cerebral hemorrhage and cerebral infarction), Parkinson’s syndrome, epilepsy, bipolar disorder, and other neuropsychiatric disorders; (5) Had severe head trauma, carbon monoxide poisoning, etc.

### 2.2. Instruments

#### 2.2.1. The general information questionnaire

The questionnaire included: age, sex, years of education, marital status, prior psychiatric history, family history of dementia, history of physical diseases, etc.

#### 2.2.2. Hopkins verbal learning test-revised

The CNCB included administration of the HVLT-R. The electronic version of the HVLT-R requires the subject to complete the test via a Pad. At the start of the test, the Pad automatically reads out the instructions in standard Mandarin, and when the subject clicks to confirm the test, the Pad starts to read out the list at a rate of one word every 2 s, and three immediate recall trials are performed. After 5 and 20 min, the Pad automatically performs the short and long-delayed recall trials. The long-delayed recall trial was followed by a recognition trial. During the learning and delayed recall trials, the screen displays 12 word buttons, with additional buttons such as “repeat,” “other word,” “not attempted,” and “undo” located below. The experimenter selects the appropriate button by clicking on it. During the recognition trial, the experimenter reads out the 24 words on the screen in sequence and selects the correct or incorrect button based on the participant’s response. The administration process of the PAP-HVLT-R can be found in Benedict et al.’s paper (Benedict et al., 1998). The same word lists were used for both PAP and Pad versions of the HVLT-R.

Variables that were directly derived from the number of correct words given in each trial were considered direct variables, while variables that were derived indirectly from ratios were considered derived variables. Table 2 shows a detailed description of each variable.

**Table 2:** Variables selected for analysis, conceptual detail.

Name	Description
**Direct**	
Total recall	The sum of correct words recalled on trial 1, 2, and 3.
Short-delayed recall	The number of correct words on trial 4.
Long-delayed recall	The number of correct words on trial 5.
Recognition discrimination index (RDI)	the number of true positives minus the number of false positives in the recognition trial.
**Derived**	
Short-delayed recall percent retained (SDR percent retained)	The number of words recalled in trial 4 divided by the higher of trials 2 or 3.
Long-delayed recall percent retained (LDR percent retained)	The number of words recalled in trial 5 divided by the higher of trials 2 or 3.
Semantic clustering (Stricker et al., 2002)	The number of times a correctly recalled word was followed by another correctly recalled word appearing in the same semantic feature was divided by the number of correct recalled per trial.
Serial clustering (Stricker et al., 2002)	The number of times a correctly recalled word was followed by another correctly recalled word appearing in the same list order divided by the number of correct recalled per trial.
Perseverations	The sum of repetition words recalled on trial 1–5.
Intrusions errors	The number of words recalled that were not HVLT-R stimuli on trial 1–5.

#### 2.2.3. Other tests

The CNCB also included the Hong Kong Brief Cognitive Test (HKBC; Chiu et al., 2018), the Brief Visuospatial Memory Test-revised (BVMT-R; Benedict et al., 1996), the Logical Memory Test (LM) of the Wechsler Adult Memory Scale-Chinese Revision (WMS-RC; Wang et al., 2015). The LM is an assessment of narrative episodic memory. We used the total score of the HKBC, the scores of total recall (sum of correct responses on three immediate recall trials) and delayed recall of the BVMT-R, and the mean scores of immediate and delayed recall of the LM in our analyses.

Several other non-verbal tests were included in the test battery but were not included in the current study. Subjects completed the non-verbal tests in-between delayed recall sessions to avoid interference.

### 2.3. Statistical analyses

Statistical analyses were conducted using SPSS 22.0. First, the reliability of the Pad-HVLT-R was evaluated using internal consistency, split-half reliability, and test–retest reliability. The Cronbach’s alpha coefficient for trial 1 to trial 5 was used to measure internal consistency, and split-half reliability was assessed by correlating the two odd-even scores (test 1 + 3 vs. test 2 + 4, test 2 + 4 vs. test 3 + 5) of Sample 1. In addition, we separately calculated the Cronbach’s alpha coefficients for the PAD and PAP data of Sample 3. The test–retest reliability was measured by calculating Pearson’s product–moment correlations between two sessions of Sample 2, and r values of 0.3, 0.5, and 0.7 represent low, medium, and high correlations, respectively (Mukaka, 2012).

The validity of the Pad-HVLT-R was examined through concurrent validity, convergent validity, and discriminant capacity. Concurrent validity was assessed by calculating the correlation coefficients between the scores of Pad-HVLT-R and the corresponding scores of HKBC, BVMT, and LM in Sample 1. Convergent validity was examined by calculating the correlation coefficients for the corresponding variables between the Pad and the PAP version of the HVLT-R in Sample 3. Discriminant capacity was examined by conducting the group comparisons and the receiver operating characteristic curve (ROC) analysis for the healthy group and case with aMCI and AD. Since previous findings have shown a significant effect of age and education on the HVLT (Shi et al., 2012; Duff, 2016; Ryan et al., 2021), in this study we used the Propensity Score Matching (PSM) in SPSS 22.0 to select two healthy groups in Sample 1 that matched the age and education of the aMCI and AD groups, respectively, to reduce the effect of age and education. In the ROC analysis, the cut-off scores were determined by the Youden index, with the highest Youden index being the optimum value for sensitivity and specificity.

In this study, the normal probability plot was used to assess whether or not the data are approximately normally distributed (Ghosh, 1996). For correlation analysis, Pearson’s correlation analysis was used for normal data and Spearman’s correlation analysis was used for non-normality. In the group comparison, the independent samples *t*-test was used for normal data and the Mann–Whitney *U*-test was used for non-normality.

## 3. Results

### 3.1. Descriptive statistics

Demographic information of the healthy sample is presented in Table 1.

### 3.2. Reliability

#### 3.2.1. Internal consistency reliability

It is calculated that the Cronbach’s α coefficient of the electronic version of HVLT-R is 0.94. The correlation coefficients of the two odd-even scores are 0.91 and 0.92, respectively, and the split-half reliability is 0.96 calculated by the Spearman-Brown formula. In addition, the electronic version had a Cronbach’s α coefficient of 0.849 and the paper-pencil version had a reliability coefficient of 0.899.

#### 3.2.2. Test–retest reliability

Table 3 presents the raw descriptive data for the electronic version of HVLT-R, along with the r values for the twice examined. The test–retest correlation coefficients were higher for the direct variables overall compared to the derived variables. The highest correlation was observed for total recall (r = 0.62) and the lowest coefficient of retest was observed for the index of RDI (r = 0.38). Whereas among the derived variables, the highest correlation was observed for semantic clustering (r = 0.52), more modest test–retest stability coefficients were observed for serial clustering, perseverations, and intrusions errors, while the correlations for the other derived measures were lower.

**Table 3:** Correlation values for the test–retest of the Pad-HVLT-R variables.

Variable	1st assessment	2nd assessment	r	*p*
**Direct**				
Total recall	20.07 (5.43)	22.86 (4.67)	0.65	<0.001
Short-delayed recall	6.40 (3.19)	8.05 (2.25)	0.54	<0.001
Long-delayed recall	6.07 (3.11)	7.81 (2.39)	0.44	0.003
RDI	8.98 (1.93)	9.91 (1.36)	0.38	0.012
**Derived**				
SDR percent retained	0.78 (0.34)	0.97 (0.26)	0.16	0.318
LDR percent retained	0.75 (0.36)	0.94 (0.27)	0.18	0.251
Semantic clustering	1.90 (0.74)	2.09 (0.79)	0.52	<0.001
Serial clustering	0.34 (0.24)	0.43 (0.30)	0.34^a^	0.029
Perseverations	5.83 (6.58)	7.24 (9.85)	0.44^a^	0.008
Intrusions errors	2.74 (3.32)	3.07 (4.26)	0.36^a^	0.019

RDI, recognition discrimination index; SDR percent retained, Short-delayed recall percent retained; LDR percent retained, Long-delayed recall percent retained; ^a^Spearman’s rank correlation coefficient.

### 3.3. Validity

#### 3.3.1. Concurrent validity

Table 4 presents the correlation coefficients between the Pad-HVLT-R and the HKBC, BVMT-R, and LM. There was a moderate correlation between the HVLT-R, HKBC, and BVMT-R. The total and long-delayed recall of the HVLT-R correlated most strongly with the corresponding variables of the LM.

**Table 4:** Pearson’s correlation between key Pad-HVLT-R indexes and other tests.

		HKBC (*n* = 1,538)	BVMT-R (*n* = 570)		LM (*n* = 1,697)	
	Variable	Total score	Total recall	Delayed recall	Immediate recall	Delayed recall
HVLT-R	Total recall	0.41^**^	0.54^***^	0.56^***^	0.72^***^	0.70^**^
	Long-delayed recall	0.42^**^	0.56^***^	0.59^***^	0.60^***^	0.62^**^

HVLT-R, the Hopkins Verbal Learning Test-Revised; HKBC, the Hong Kong brief cognitive test; BVMT-R, the Brief visual memory test-revised; LM, the logical memory Test; ^**^p < 0.01; ^***^p < 0.001.

#### 3.3.2. Convergent validity

Correlation analysis between the PAP and electronic versions of the HVLT-R showed that the correlation coefficients for all direct variables were significant, with only the correlation coefficient for the RDI being slightly below 0.30, while among the derived variables only the LDR percent retained and semantic clustering were above 0.30 (Table 5).

**Table 5:** Correlation analysis of the PAP-HVLT-R with the Pad-HVLT-R.

Variable	PAP-HVLT-R	Pad-HVLT-R	r	*p*
**Direct**				
Total recall	18.08 (4.61)	20.08 (4.93)	0.53	<0.001
Short-delayed recall	6.06 (2.30)	6.66 (3.15)	0.48	<0.001
Long-delayed recall	6.12 (2.44)	6.34 (3.11)	0.55	<0.001
RDI	8.72 (1.72)	8.43 (2.32)	0.29	0.020
**Derived**				
SDR percent retained	0.81 (0.33)	0.79 (0.34)	0.15	0.228
LDR percent retained	0.81 (0.32)	0.75 (0.34)	0.31	0.012
Semantic clustering	1.63 (0.64)	1.93 (0.84)	0.43	<0.001
Serial clustering	0.33 (0.21)	0.47 (0.38)	0.25^b^	0.049
Perseverations	2.15 (2.59)	7.57 (14.00)	0.12^b^	0.356
Intrusions errors	4.09 (3.98)	3.15 (4.46)	0.28^b^	0.025

PAP-HVLT-R, the paper-and-pencil version of the HVLT-R; Pad-HVLT-R, the electronic version of the HVLT-R; RDI, Recognition discrimination index; SDR percent retained, Short-delayed recall percent retained; LDR percent retained, Long-delayed recall percent retained; ^b^Spearman’s correlation coefficient for ranked data.

#### 3.3.3. Discriminant capacity

We rigorously compared the performance of the Pad-HVLT-R between HC and aMCI and AD groups. The results showed no significant differences in age, education, and sex ratio between the HC group and the aMCI and AD groups after automatic matching by PSM.

The results of group comparisons showed that the HC group had significantly higher scores than the aMCI group on all direct variables as well as on the long-delayed recall percent retained, semantic cluster ratio, and perseverations of the derived variable, while there were no significant differences between the two groups on other variables. The results of the comparison are shown in Table 6.

**Table 6:** Comparison of demographic data and Pad-HVLT-R scores of the HC and aMCI group.

	HC (*n* = 45)	aMCI (*n* = 45)	HC vs. aMCI	
Variable	M (SD)	M (SD)	*t*/*χ*^2^	*p*
Age group	60.64 (15.04)	60.76 (14.69)	−0.038	0.969
Education	10.22 (3.60)	10.37 (4.10)	−0.185	0.853
Sex (male/female)	18/27	22/23	0.720	0.396
**Direct**				
Total recall	22.04 (5.95)	16.67 (6.74)	4.012	<0.001
Short-delayed recall	7.84 (3.23)	5.47 (3.16)	3.529	0.001
Long-delayed recall	7.47 (3.19)	4.89 (3.26)	3.795	<0.001
RDI	9.31 (2.32)	7.56 (2.45)	3.484	0.001
**Derived**				
SDR percent retained	0.88 (0.27)	0.77 (0.39)	1.677	0.097
LDR percent retained	0.84 (0.32)	0.67 (0.39)	2.392	0.019
Semantic clustering	2.02 (0.70)	1.32 (0.76)	4.507	<0.001
Serial clustering	0.39 (0.34)	0.41 (0.37)	−0.040^c^	0.968
Perseverations	4.07 (5.01)	2.53 (5.12)	−2.045^c^	0.041
Intrusions errors	2.96 (4.30)	2.53 (5.73)	−1.273^c^	0.203

HC, healthy control; aMCI: amnestic Mild Cognitive Impairment; RDI, recognition discrimination index; SDR percent retained, Short-delayed recall percent retained; LDR percent retained, Long-delayed recall percent retained; ^c^Mann-Whitney *U*-test.

The results for the AD and the HC group showed that the HC group scored significantly higher than the AD group on all variables except intrusions errors, the results of which are shown in Table 7.

**Table 7:** Comparison of demographic data and Pad-HVLT-R scores of the HC and AD group.

	HC (*n* = 42)	AD (*n* = 42)	HC vs. AD	
	M (SD)	M (SD)	*t*/*χ*^2^	*p*
Age group	68.35 (11.04)	72.26 (8.41)	−1.847	0.068
Education	8.42 (4.59)	8.19 (4.37)	0.236	0.814
Sex (male/female)	24/19	20/23	0.745	0.388
**Direct**				
Total recall	21.07 (9.74)	8.95 (5.44)	7.119	<0.001
Short-delayed recall	6.93 (3.34)	1.07 (1.98)	9.892	<0.001
Long-delayed recall	6.35 (2.95)	0.88 (1.83)	10.323	<0.001
RDI	8.02 (2.83)	2.47 (2.77)	9.192	<0.001
**Derived**				
SDR percent retained	0.80 (0.23)	0.16 (0.25)	12.148	<0.001
LDR percent retained	0.74 (0.22)	0.15 (0.3)	10.380	<0.001
Semantic clustering	1.24 (0.58)	0.67 (0.51)	4.871	<0.001
Serial clustering	0.74 (0.59)	0.22 (0.27)	−4.748 ^c^	<0.001
Perseverations	5.26 (6.71)	0.91 (1.41)	−4.775 ^c^	<0.001
Intrusions errors	3.05 (3.98)	1.77 (2.22)	−1.537 ^c^	0.124

HC, healthy control; AD, Alzheimer’s disease; RDI, recognition discrimination index; SDR percent retained, Short-delayed recall percent retained; LDR percent retained, Long-delayed recall percent retained; ^c^Mann-Whitney *U*-test.

To further determine the discriminant ability of the Pad-HVLT-R to detect aMCI and AD patients from the healthy population, we calculated the AUC which is typically rated as acceptable (0.70–0.79), good (0.80–0.89), excellent (0.90–0.99) or perfect (1.0; Carter et al., 2016). In distinguishing aMCI and HC, the AUC of all variables was higher than 0.70, among which the AUC of the total recall was the highest, and the optimal sensitivity and specificity were achieved when the cut-off score was 21.50 (out of a maximum score of 36). When distinguishing between AD and HC groups, the AUC was higher than 0.90 for all variables except the total recall. The best sensitivity and specificity were achieved when the cut-off score for the total recall was 16.50. The optimal sensitivity and specificity were achieved when the cut-off score of the long-delayed recall was 1.50 (out of a maximum score of 12). Table 8 shows the AUC, cut-off score, sensitivity, and specificity of the direct variables of the Pad-HVLT-R for the aMCI and AD patients, respectively.

**Table 8:** Results of ROC analysis between HC with aMCI and AD group.

Var.	AUC	*p*	Cut-off	SE	SP
**HC vs. aMCI**					
Total recall	0.726	<0.001	21.50	0.756	0.600
Short-delayed recall	0.701	0.001	7.50	0.733	0.556
Long-delayed recall	0.723	<0.001	5.50	0.578	0.778
RDI	0.703	0.001	9.50	0.800	0.556
**HC vs. AD**					
Total recall	0.834	<0.001	16.50	0.953	0.628
Short-delayed recall	0.923	<0.001	2.50	0.814	0.860
Long-delayed recall	0.934	<0.001	1.50	0.791	0.930
RDI	0.906	<0.001	5.50	0.814	0.837

HC, healthy control; aMCI: amnestic Mild Cognitive Impairment; AD, Alzheimer’s disease; AUC, area under the curve; SE, sensitivity; SP, specificity; RDI, recognition discrimination index.

## 4. Discussion

In this study, we examined the reliability and validity of the electronic version of the Hopkins Verbal Learning Test-Revised in middle-aged and elderly Chinese people, and the results showed that the Pad-HVLT-R had good reliability and validity. In clinical samples, the results showed that the Pad-HVLT-R was effective in assessing verbal memory function and its impairment, and sensitive to changes in memory ability in AD patients, but not as effective in differentiating between aMCI patients and the healthy population.

The Cronbach’s alpha was 0.94 and the split-half reliability was 0.96, indicating good internal consistency reliability of the electronic version of HVLT-R. Although the Cronbach’s α coefficient of the electronic version was slightly lower than that of the paper-pencil version calculated in Sample 3, both versions still exceeded the recommended threshold of 0.70 for internal consistency reliability. Therefore, the findings suggest that the electronic HVLT-R is a reliable tool for episodic memory assessment. The test–retest reliability coefficients of the direct variables were relatively high, with total recall and short-delayed recall being the highest. The results are broadly similar to those described by Barr (2003) and the work of Woods et al. (2005) but lower than those reported by Benedict et al. (1998), which is presumably related to the sample size or the interval between two examine (e.g., the three studies mentioned above had retest intervals of 60, 370, and 47 days). However, the correlation coefficients for some derived variables were relatively low or even negative, except for semantic clustering. Therefore, it is possible to be cautious in interpreting changes in the derived variables of the HVLT-R over time (Woods et al., 2006). Overall, the test–retest reliability of the electronic version of the HVLT-R direct variables is moderate, but of some derived variables is low.

The concurrent validity was assessed by calculating the correlation coefficients of the Pad-HVLT-R with the HKBC, the BVMT-R and the LM, respectively. Scores on the HVLT-R correlated most strongly with the LM scores. The HVLT-R also correlated strongly with the BVMT-R but more modestly correlated with the HKBC. Notably, the LM and the BVMT-R used in this study were administered in electronic format, and their reliability and validity have not yet been established, which could potentially impact the correlations between the tests. Overall, the findings provide sufficient evidence that the electronic version of the HVLT-R has concurrent validity to justify its use in clinical neuropsychological assessments.

Regarding convergent validity, we used a crossover design to control for possible order effects. The results showed moderately significant correlations between the direct variables, with the exception of the recognition discrimination index. In contrast, only moderate correlation coefficients were found between long-delayed recall percent retained and semantic clustering among the derived variables, while all others were relatively low. Due to practical constraints, this part of the psychometric analysis only included healthy participants, which may have led to a restricted range of scores on the variables being measured and could have influenced the results of the convergent validity analysis. Additionally, measurement error may have been introduced due to differences between the two test formats, as well as differences in the context of the two administrations and the time interval between the two administrations, which may result in lower correlation coefficients. In addition, we compared the correlation coefficients for the PAP and Pad with the test–retest coefficients for the Pad-HVLT-R, found that the two were similar and that the correlation coefficients for the PAP and Pad format were lower than the test–retest coefficients except for the perseverations, which maybe since the variations in scores between PAP and Pad came not only from random variation but also from variation in the construct between assessments. The lower number of perseverations on the PAP compared to the Pad version may be because of the fact that the electronic version is more convenient to record, allowing for a quick recording of all of the subject’s responses without omission.

We evaluated the capacity of the electronic version of the HVLT-R to distinguishing aMCI and AD cases from HC group. The results showed significant differences between participants in the impaired groups (aMCI and AD) and healthy controls in all direct variables of the Pad-HVLT-R. As for the derived variables, there were significant differences between the impaired and HC groups except for intrusions errors. All direct variables and most derived variables of the Pad-HVLT-R were able to separate older adults with AD or aMCI from healthy individuals.

When detecting AD from HC, the AUC for all direct variables except total recall were higher than 0.90. The optimal balance between sensitivity and specificity was achieved when the cut-off score for long-delayed recall was 1.50, at which point the specificity was good (0.930) and the sensitivity was moderate (0.791). Compared with other variables, the AUC of total recall (0.811) was slightly lower, with high sensitivity (0.953) and lower specificity (0.628) when the optimal demarcation was 16.50. Overall, the electronic version of the HVLT-R is good at distinguishing between AD and HC.

However, the results showed a relatively low discrimination capacity of the Pad-HVLT-R to detecting aMCI cases from HC group. The AUC for total recall was highest, and the optimal balance between sensitivity (75.6%) and specificity (60.0%) with the total recall score was at a cut-off point of 21.50. The most optimal cut-off scores of the total recall for detecting aMCI and AD from healthy subjects in this study are similar to the results of Shi et al. (2012). They examined the discriminant capacity of the Chinese version of HVLT for MCI and dementia and showed that the optimal cut-off score for discriminating aMCI from the HC group was 21.50, when the sensitivity and specificity were lower (69.1% and 70.7%), whereas the sensitivity and specificity for distinguishing AD from the HC group was high when the cut-off score was 15.50. The results of the present study are broadly similar to those of previous studies on the discriminant capacity of the PAP-HVLT-R for AD and aMCI, which showed high discrimination for AD and relatively lower discrimination for aMCI (de Jager et al., 2003; Schrijnemaekers et al., 2006; Shi et al., 2012).

There are some limitations in this study: (1) There was a design flaw in the computerized test system upfront, leading to the loss of some data due to the failure of a few subjects to complete delayed recall, which has now been corrected; (2) The sample size of AD and aMCI cases included in this study was small and their education level was high (73% of aMCI patients had more than 9 years of education), which limited the follow-up analysis of different ages and education levels; (3) Due to practical constraints, we did not collect data on patients’ performance on the PAP version. This may have affected the results of the convergent validity analysis. In future studies examining the convergent validity of the electronic test, both healthy and clinical participants could be included to complete the PAP and Pad versions of the test, in order to improve the generalizability of the results.

In conclusion, the electronic version of the Hopkins Verbal Learning Test-Revised has good reliability and validity, and can be used in clinical and basic research to assess the verbal memory function of middle-aged and older Chinese people.

## Data availability statement

The raw data supporting the conclusions of this article will be made available by the authors, without undue reservation.

## Ethics statement

The studies involving human participants were reviewed and approved by the Second Xiangya Hospital of Central South University Ethics Committee. The patients/participants provided their written informed consent to participate in this study.

## Author contributions

DW and CS designed this study. LJ and DW analyzed the data and prepared the manuscript. All authors contributed to the article and approved the submitted version.

## Funding

This work was supported by the National Key R&D Program of China (2018YFC1314202).

## Conflict of interest

The authors declare that the research was conducted in the absence of any commercial or financial relationships that could be construed as a potential conflict of interest.

## Publisher’s note

All claims expressed in this article are solely those of the authors and do not necessarily represent those of their affiliated organizations, or those of the publisher, the editors and the reviewers. Any product that may be evaluated in this article, or claim that may be made by its manufacturer, is not guaranteed or endorsed by the publisher.
